# Overexpression of *OsEXPA8*, a Root-Specific Gene, Improves Rice Growth and Root System Architecture by Facilitating Cell Extension

**DOI:** 10.1371/journal.pone.0075997

**Published:** 2013-10-04

**Authors:** Nana Ma, Ying Wang, Shichun Qiu, Zhenhui Kang, Shugang Che, Guixue Wang, Junli Huang

**Affiliations:** Bioengineering College, Chongqing University, Chongqing, China; University of Nottingham, United Kingdom

## Abstract

Expansins are unique plant cell wall proteins that are involved in cell wall modifications underlying many plant developmental processes. In this work, we investigated the possible biological role of the root-specific α-expansin gene *OsEXPA8* in rice growth and development by generating transgenic plants. Overexpression of *OsEXPA8* in rice plants yielded pleiotropic phenotypes of improved root system architecture (longer primary roots, more lateral roots and root hairs), increased plant height, enhanced leaf number and enlarged leaf size. Further study indicated that the average cell length in both leaf and root vascular bundles was enhanced, and the cell growth in suspension cultures was increased, which revealed the cellular basis for *OsEXPA8*-mediated rice plant growth acceleration. Expansins are thought to be a key factor required for cell enlargement and wall loosening. Atomic force microscopy (AFM) technology revealed that average wall stiffness values for *35S*::*OsEXPA8* transgenic suspension-cultured cells decreased over six-fold compared to wild-type counterparts during different growth phases. Moreover, a prominent change in the wall polymer composition of suspension cells was observed, and Fourier-transform infrared (FTIR) spectra revealed a relative increase in the ratios of the polysaccharide/lignin content in cell wall compositions of *OsEXPA8* overexpressors. These results support a role for expansins in cell expansion and plant growth.

## Introduction

Plant growth and development depends on the regulation of cell extension and growth through cell wall modifications such as loosening and reassembly. Proteins in the cell wall are believed to play important roles in the modulation of cell wall extensibility, a crucial parameter in determining cell expansion [1]. Expansins are thought to be a key wall-loosening factor that have long been implicated in the control of plant growth processes via their role as modulators of cell wall extensibility [2], although the exact mechanism of expansin activity remains unclear. Experimental evidence suggests that they may disrupt the non-covalent bonding between the cellulose microfibrils and matrix polysaccharides, therefore permitting the loosening and extension of the cell wall and the turgor-driven growth of the cell [3], [4].

There are two expansin families (EXPA and EXPB) that facilitate cell wall stiffness relaxation and cell extension. A number of investigations have been performed to isolate and express expansin genes, but to date only limited information is available to elucidate their precise biological functions during plant development. *LeEXP1* is demonstrated to be involved in tomato fruit softening by overexpression and suppression strategies [5], [6]. Transient local microinduction of *CsEXP1* in the shoot apical meristem of *Nicotiana tabacum* induces the leaf development process, leading to altered local lamina and abnormal leaves [7]. Transgenic tobacco plants with constitutive expression of *ClEXPA1* and *ClEXPA2* from *Cunninghamia lanceolata* display growth and development alteration and the amount of cellulose increase in stem cell walls [8]. Up- and down-regulation *PhEXPA1* from *Petunia hybrida* leads to changes of cell wall polymer compositions and affects the timing of axillary meristem development in *Petunia hybrid* [1], [9]. In Arabidopsis, several expansins have been extensively studied. *AtEXPA7* and *AtEXPA18* are closely associated with root hair initiation [10], [11], while modification of *AtEXP10* expression modulates the leaf growth and pedicel abscission [11]. In addition, *AtEXPA1* accelerates stomatal opening by decreasing the volumetric elastic modulus [12].

Despite the low sequence identity, α- and β-expansins have similar rheological effect on cell walls (induction of creep and stress relaxation) [13]. It has been reported α-expansins work well on cell walls in dicots and monocots of the *Amaryllidaceae* principally, but to have relatively low effect on cell walls of graminaceous monocots. In contrast, β-expansins have more strong selectivity for cell walls of graminaceous monocots and only marginal effect was found on dicot cell walls [14]. In fact, both groups of plants have genes for both types of expansins. In a case of rice, there are at least 34 α-expansin genes, and what contributions do a large number of α-expansin genes make to rice growth and development? Clearly, understanding what and how α-expansins function during plant morphogenesis is a question of both practical and biological significance. Thus far, *OsEXP4* has been testified to be closely correlated with plant growth by sense and antisense approaches [15], and investigations of rice α-expansin defective mutants demonstrated that *OsEXPA17* and *OsEXPA30* play a crucial role in root hair elongation [16]. However the biological functions of most of the α-expansin genes in rice remain unclear. *OsEXPA8* has been reported to display root-unique expression pattern: exclusively in the first 0.5-cm region from the root tip of one-week-old seedlings and is closely related to *OsEXPA3* and *OsEXPA9* in phylogenesis [17], whereas there is limited data available to elucidate the biological function of *OsEXPA8*. In this study, we probed the effect of *OsEXPA8* on rice growth and root system architecture by regulation of *OsEXPA8* expression. Our results support current knowledge regarding universal functions of expansins and provide further evidence that endogenous OsEXPA8 plays an important role in mediating the cell extension and growth both biochemically and mechanically.

## Materials and Methods

### 1. Plant Materials and Growth Conditions

Rice (*Oryza sativa* L. cv Zhonghua 11) was used for all physiological experiments and genetic transformation. All transgenic and wild-type plants were cultivated in a greenhouse under a 12 h photoperiod (300 µmol photons m^−2^ s^−1^) and at 30°C. Humidity was controlled at approximately 60%.

### 2. Plasmid Construction and Rice Transformation

The full-length cDNA clone of *OsEXPA8* (accession number: AK111100) was obtained from the National Institute of Agrobiological Sciences (NIAS, Tsukuba, Japan). For overexpression in rice, the complete open reading frame (ORF) of *OsEXPA8* was obtained from the clone 002-175-G12 by digestion with restriction enzymes *Kpn* I and *Pst* I, and cloned into the modified pCAMBIA1301 to generate *35S::OsEXPA8*, which contained a hygromycin-resistance selectable marker system and a GUS reporter system.

The construct *35S::OsEXPA8* was transformed into rice calli according to the genetic transformation method as described by Toki et al. [18]. Transgenic status of *35S::OsEXPA8* transformants was confirmed by hygromycin-resistance and GUS staining, and further mRNA levels of *OsEXPA8* were determined by quantitative real-time PCR.

### 3. Expression Analysis of *OsEXPA8* in Transgenic Rice Plants

Quantitative real-time PCR was employed to analyze the expression levels of *OsEXPA8* in transgenic plants using specific primers (Table 1) with *β-actin* as the internal control [19]. Total RNA was isolated from rice leaf samples with TRIZOL reagent (Invitrogen, USA), and RNA quality and quantity was determined by electrophoresis and spectrophotometry. Total RNA from each sample was reverse transcribed and quantitative real-time PCR was performed in a CFX96 thermal cycler (Bio-Rad, USA) using SYBR® *Premix Ex Taq*™ II (Takara, Japan). The expression levels, normalized to that of *β-actin*, were calculated by relating the measured Ct to a standard curve.

**Table 1:** Primers used in this study.

Names	Sequences
*Os EXPA8q-*Fw	5′-GCGATGAGCCGCAACTG-3′
*Os EXPA8q-*Rv	5′-CTCTTCCATCCTATGCCACG-3′
*β-actin-*Fw	5′-AGGAAGGCTGGAAGAGGACC
*β-actin-*Rv	5′-CGGGAAATTGTGAGGGACAT

Fw, forward primers; Rv, reverse primers.

### 4. Measurement of Morphological and Physiological Properties of Transgenic Plants, Microtechniques and Microscopy

To check the effect of *OsEXPA8* overexpression on plant growth and development, the plant height was measured from the base of the stem to the top of the flag leaf. For root hair epidermal observation, seeds were surface-sterilized for 10 min in 1% sodium hypochlorite and thoroughly washed three times with sterile distilled water. The seeds were placed on half-strength MS solid medium and grown for 7 days vertically in a growth chamber. Stereoscope and cryo-scanning electron microscope (Cryo-SEM) images were acquired at the region 3–5 mm from the root apex. Stereoscope images were acquired by a stereoscopic microscope (Olympus, Japan). Cryo-SEM was performed as described previously [20] and root hairs were observed using a Hitachi S-3000N scanning electron microscope (Hitachi, Japan).

The flag leaf and lateral root were used for assessing the leaf length and width, and also for transverse and longitudinal sections. Fresh leaf or root samples were fixed overnight in 50% FAA (50% ethyl alcohol: glacial: formaldehyde acetic acid = 16∶1:1). Samples were then dehydrated in a graded ethanol series, embedded in paraffin wax, and sectioned with a LKB ultramicrotome. Sections (4–7 µm) were dried onto slides at 37°C and stained for microscopic observation, and section microscopic images were acquired with an optical microscope (Olympus, Japan).

### 5. Rice Suspension-cultured Cells

Rice suspension cells were cultured using the calli according to the procedure reported previously [21] with minor modification. Friable calli were selected and transferred to initiate cell suspension cultures in liquid AA medium supplemented with 2 mg/L of 2, 4-dichlorophenoxyacetic acid (2, 4-D) and 3% of sucrose. The suspension cultures were incubated at 28°C under conditions of continuous dark and constant agitation (140 rpm), and subcultured every 7 days by transferring 20 ml of suspensions to 200 ml of fresh medium. After at least four successive subculturing cycles, the cells were sampled for measuring growth characteristics and the growth medium was used for measuring pH value on days 3, 5, 7, 10 and 13, and the cell wall stiffness was measured on days 5, 10 and 13.

The suspension cultures were centrifuged at 2000×*g* and the precipitate was quantified for weight before transferred to fresh medium. The growth rate of suspension cells were determined by measuring the relative growth increment per day using the formula P =  (W1−W0)/W0/culture time (d) ×100%. P: relative growth rate; W1: weight of cells collected by vacuum filtration at each sampling juncture; W0: initial weight of cells before transferred to medium.

### 6. Measurement of the Elastic (Young’s) Modulus of the Cell Wall by Atomic Force Microscopy (AFM) Technology

**Figure 1:**
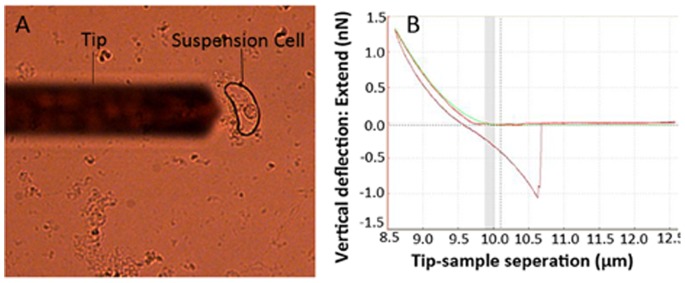
Stiffness-properties imaging by atomic force microscopy (AFM) technology. (A) Optical image of a typical single rice cell sample. The shadow of the AFM cantilever is visible on the left-hand side of the image. (B) A typical force-distance (FD) curve recorded on a single rice cell sample. The upper line represents the FD curve when the tip indents (or penetrates) the cell wall and the lower line represents the FD curve when the tip retracts from the cell wall.

Measurement of the elastic modulus of the cell wall was performed according to the recent report [22] with minor modification. In our study, AFM analysis was performed in microscope (JPK, Germany). The rice cell cultures, suspended in growth medium, were deposited upon glass petri dish covers that had been pretreated with poly-l-lysine (0.1 mg ml^−1^), and cultured at 28°C overnight for adhering of cells. For the analysis, we used n-type silicon pyramid cantilevers with a semi-opening angle of 20° on average and a nominal spring constant of 0.3 N m^−1^ (Umasch, Russia, http://www.spmtips.com). The precise spring constant of each cantilever was verified using the *JPK image processing* software, which is implemented in JPK microscopes. Figure 1A demonstrated a single rice cell sample under the AFM cantilever tip. The tips we used in this experiment had a nominal radius of curvature of 8 nm. The deflection sensitivity was determined before the experiments by recording a set of force-distance (FD) curves over the petri dish cover. FD curves were successively recorded over the surface of the sample using the force-volume mapping mode. The force-volume files were processed to obtain the stiffness values (elastic moduli), which are an important cell wall feature. Figure 1B displayed a typical FD curve recorded on a single rice cell sample. An FD curve monitors the deformation of the cantilever as its tip indents (or penetrates) the sample. All AFM measurements were performed on living single cells in liquid. For each sample, at least 30 cells were measured for evaluating the cell wall stiffness, and five FD curves were achieved on each cell. The Hertz model in *JPK image processing* software was used to extract stiffness property data from the native force-volume files.

### 7. Preparation of Cell Wall Isolates from Suspension Cells and Fourier-transform Infrared (FTIR) Spectroscopy

Suspension-cultured cells were collected on day 10 by vacuum filtration and homogenized in 50 mM potassium phosphate buffer (pH 7.0), and cell walls were extracted according to the methodology described [23], [24] with minor modification. This involved a resuspension and 30-min wash of the pellet in 1 M NaCl, then in 0.5% Triton X-100, and three washes first in distilled water, then in 100% methanol, and finally in 100% acetone. The isolated cell walls were then dried in a vacuum for FTIR spectroscopy.

The FTIR spectra of the isolated cell walls were recorded in transmittance mode with the KBr pellet technique, using Lambda 900 Spectrum GX FTIR Spectrophotometer (Perkin Elmer, USA).

### 8. Statistical Analysis

All measurement data were subjected to statistical analysis using the Student’s *t*-test program. The quantitative differences between two groups of data for comparison in all these experiments were shown to be statistically significant (*P*<0.01).

## Results

### 1. Overexpression of *OsEXPA8* in Rice Plants Yields Pleiotropic Phenotypes

**Figure 2:**
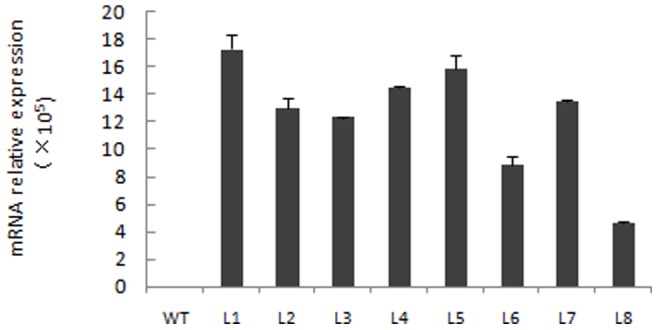
mRNA level analyses for *OsEXPA8* by quantitative real-time PCR in *35S::OsEXPA8* transgenic lines. Mature leaves of eight independent adult transgenic lines harboring *35S::OsEXPA8* and wild-type (WT) rice plants were used for investigating *OsEXPA8* expression. L1–L8 (line 1 to line 8) represents eight independent transgenic lines. Values are the means of three biological replications ± standard error. One independent transgenic plant was considered as one biological replication.

To determine the role of endogenous expansins in growth and development of rice plants, we transformed rice embryogenic calli with the construct *35S::OsEXPA8*. A total of eight independent regenerants ectopically expressing *OsEXPA8* were obtained. The presence of the transgene was confirmed by GUS staining and PCR (data not shown). Further, the expression levels of the transgene were evaluated by quantitative real-time PCR performed with total RNA extracted from the mature leaves of eight transgenic plants. As an exclusively expressed gene in rice root, *OsEXPA8* does not express in leaf and other tissues [17]; therefore, the leaf is suitable for determining the expression of the introduced *OsEXPA8* construct. As shown in Figure 2, ectopically overexpression of *OsEXPA8* in rice leaf led to *OsEXPA8* mRNA levels to increase by over 10^5^- fold in transgenic lines compared to wild-type plants. All *35S::OsEXPA8* transgenic lines displayed similar morphological phenotypes (data not shown).

**Figure 3:**
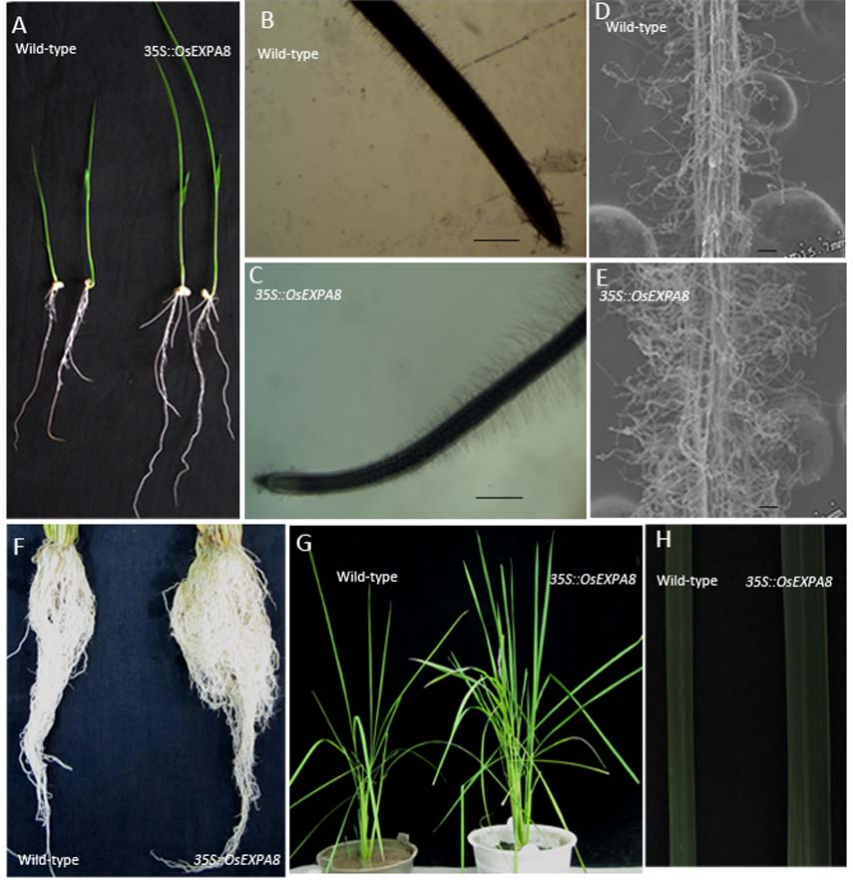
Morphological changes in *35S*::*OsEXPA8* transgenic lines. (A) Seven-day-old rice seedlings of wild-type and *35S::OsEXPA8* transgenic line1 plants. (B, C) Stereoscope images of the primary root of wild-type (B) and *35S::OsEXPA8* line1 (C) seedlings. Scale bars = 40 µm. (D, E) Epidermal morphology of the root hair growth zones of seminal roots of wild-type (D) and *35S::OsEXPA8* line1 (E) seedlings. Scale bars = 10 µm. Seedlings were grown for 7 days on half-strength solid MS medium vertically, and the root apex of was observed by stereoscope microscopes and cryo-scanning electron microscope (Cryo-SEM), respectively. (F) Root system architecture of two-month-old wild-type and *35S::OsEXPA8* line1 plants. (G) The plant morphology of two-month-old wild-type and *35S::OsEXPA8* line1 plants. (H) Flag leaf phenotype of two-month-old wild-type and *35S::OsEXPA8* line1 plants.

**Figure 4:**
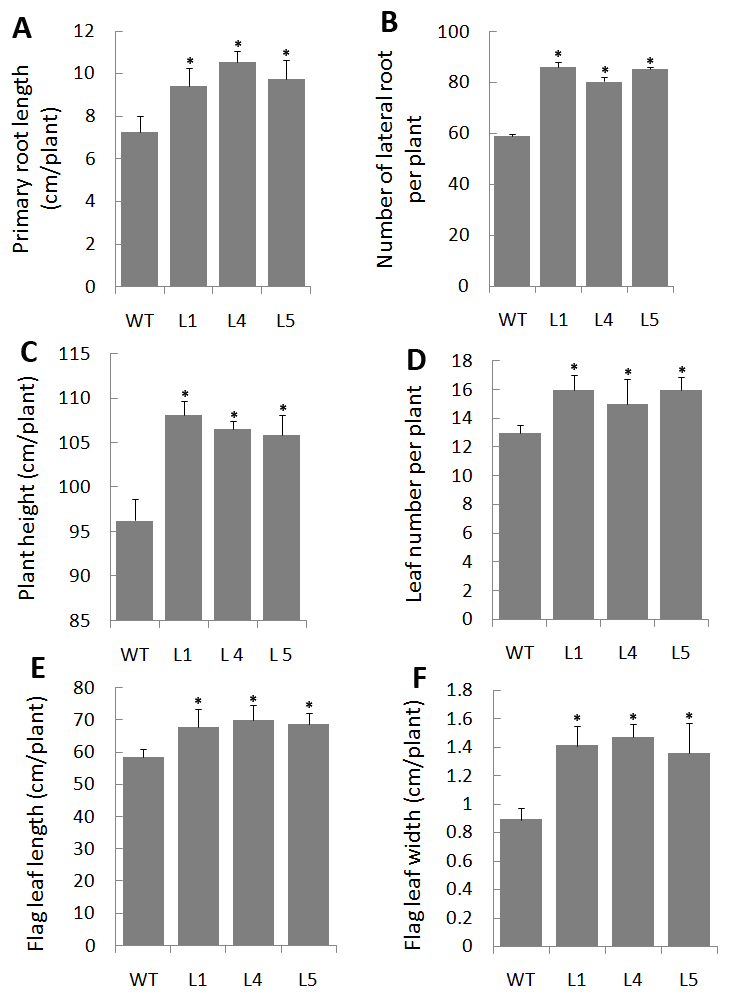
Effect of overexpression of *OsEXPA8* on the root system architecture and plant growth. (A) The length of primary roots of seven-day-old rice seedlings. (B) The number of lateral roots of seven-day-old rice seedlings. (C) The plant height of two -month-old rice plants. (D) The leaf number per plant of two-month-old rice plants. (E) The length of the flag leaf of two-month-old rice plants. (F) The width of the flag leaf of two-month-old rice plants. Three independent transgenic line 1 (L1), line 4 (L4) and line 5 (L5) were analyzed. WT: wild-type. Values are the means of ten biological replications ± standard error. One independent plant was considered as one biological replication. Asterisks (*) indicate parameters of *35S::OsEXPA8* transgenic plants were significantly different from that of wild-type plants by statistical analysis using the Student’s *t*-test program (*P*<0.01).

Overexpression of *OsEXPA8* yielded pleiotropic phenotypes in the root system architecture (Figure 3A–F), plant height, leaf number and leaf size (Figure 3G and H). For the root parameters, we investigated the length of primary roots, number of lateral roots with the seven-day-old seedlings. Compared to wild-type plants, *35S::OsEXPA8* transgenic lines showed similar morphological phenotypes: the length of primary roots (Figure 3A, Figure 4A) and number of lateral roots (Figure 4B) were significantly increased (*t*-tes*t*, *P*<0.01). The size of root hairs in the root apex of *35S::OsEXPA8* plants has been increased clearly by stereoscope (Figure 3B and C) and Cryo-SEM images (Figure 3D and E). At maturity, the difference of the root system architecture between transgenic lines and wild-type plants was also significant, and transgenic lines developed more and longer roots (including lateral and adventurous roots) (Figure 3F). Compared with wild-type plants at maturity, the average plant height and leaf number per plant of transgenic lines increased drastically (*t*-test, *P*<0.01) (Figure 3G, Figure 4C and D). Wild-type plants had an average height of 96.27 cm and developed 13 leaves; whereas *35S::OsEXPA8* transgenic lines had an average height of 106.86 cm and developed three more leaves (Figure 4C and D). Besides, the average length and width of the flag leaf of *35S::OsEXPA8* lines were 68.92 cm and 1.41 cm, which were increased significantly compared to that of wild-type plants (leaf length of 58.52 cm and width of 0.89 cm) (Figure 3H, Figure 4E and F). This result indicated that overexpression of *OsEXPA8* is responsible for altered morphology of transgenic rice plants.

### 2. Accumulation of *OsEXPA8* in Cell Wall Enlarges Cell Size

**Figure 5:**
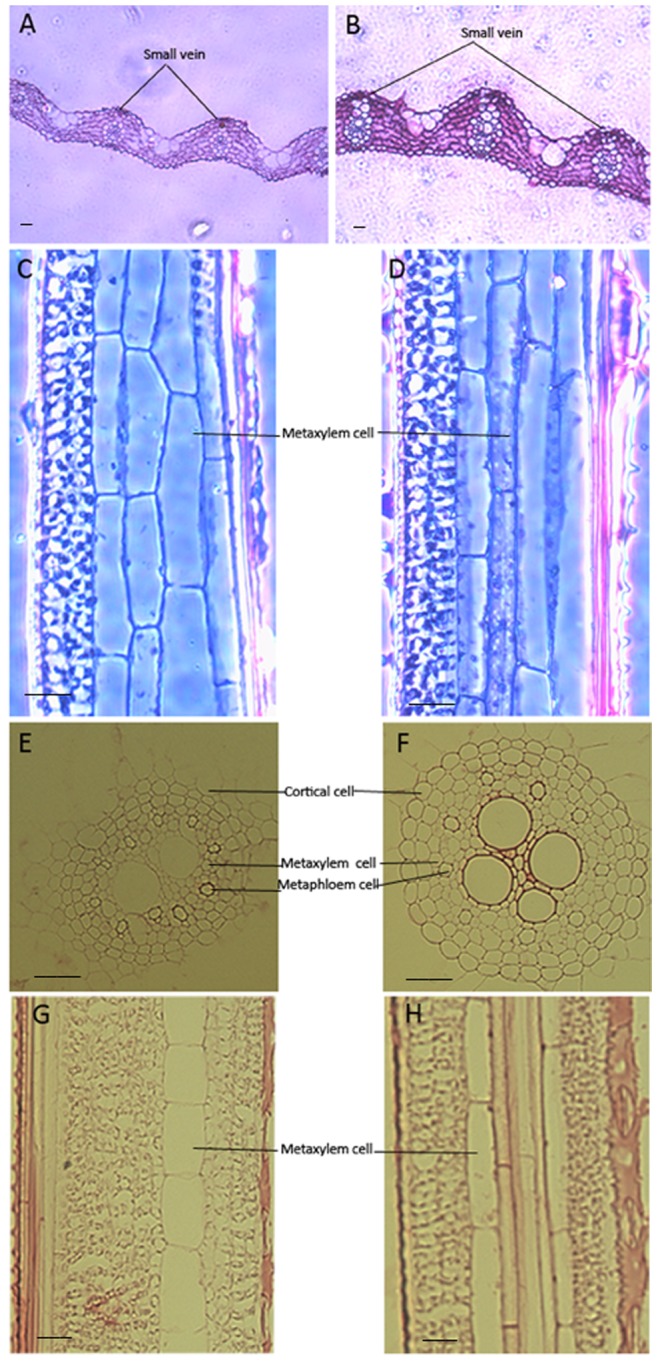
Morphological changes of vascular bundle cells in both leaves and roots in rice *35S*::*OsEXPA8* transgenic line 1 plants . Plants of two-month-old were subject to anatomic analysis. The cells were analyzed in an optical microscope. (A), (C), (E) and (G) wild-type plants; (B), (D), (F) and (H) *35S::OsEXPA8* transgenic lines; (A) and (B) transverse sections of the flag leaf, (C) and (D) longitudinal sections of the flag leaf; (E) and (F) transverse sections of the lateral root, (G) and (H) longitudinal sections of the lateral root. Scale bars: 25 µm.

**Figure 6:**
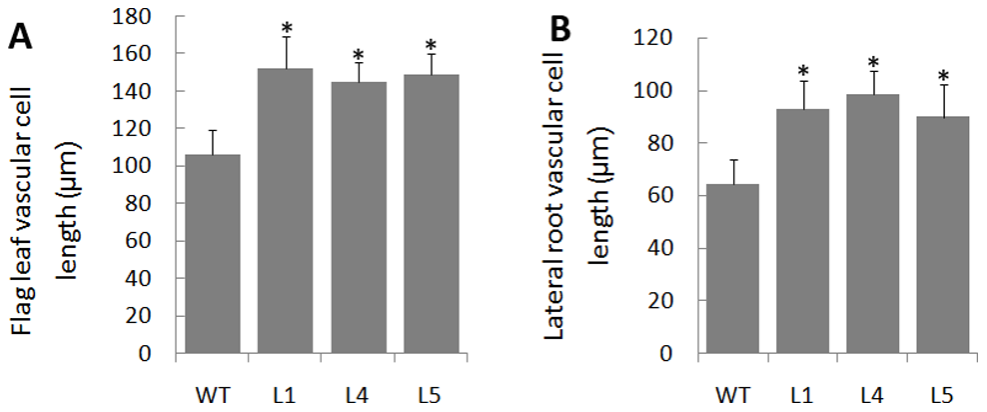
Effect of *OsEXPA8* overexpression on the cell length of vascular bundles in the flag leaf and lateral root. (A) The cell length of flag leaf vascular bundles. (B) The cell length of lateral root vascular bundles. Three independent transgenic line 1 (L1), line 4 (L4) and line 5 (L5) were analyzed. WT: wild-type. n = 100 for metaxylem cells from ten plants. Values are the means of 10 biological replications ± standard error. Asterisks (*) indicate the cell parameters of *35S::OsEXPA8* transgenic lines were significantly different from that of wild-type cells (*t*-test, *P*<0.01).

Morphological changes in plants are found to be correlated with an enlargement of cell size [8], [9]. To determine whether the enhanced growth observed in the leaf and root of *OsEXPA8* overexpression transgenic plants is correlated with cell size enlargement, we prepared both transverse and longitudinal sections of the flag leaf and lateral root from *35S::OsEXPA8* lines, and examined them by optical microscope. As we have expected, the cell size of leaf and root vascular bundles in *OsEXPA8* overexpressors was significantly larger than those of their counterparts in wild-type plants (Figure 5A–H). The average cell length of vascular bundles in the leaf in transgenic lines showed a dramatic increase (39.62%) compared to their wild-type counterparts (*t-test*, *P*<0.01) (Figure 6A). Measurement of the cell size of vascular bundles in the root was also proved that *OsEXPA8* overexpression resulted in an increased expansion of the root cell length (a 45.31% increase) (*t-test*, *P*<0.01) (Figure 6B). This result seems to be a very strong evidence for understanding the cellular basis for expansin-mediated plant growth acceleration.

### 3. The Growth of Suspension-cultured Cells is Significantly Increased by *OsEXPA8* Overexpression

**Figure 7:**
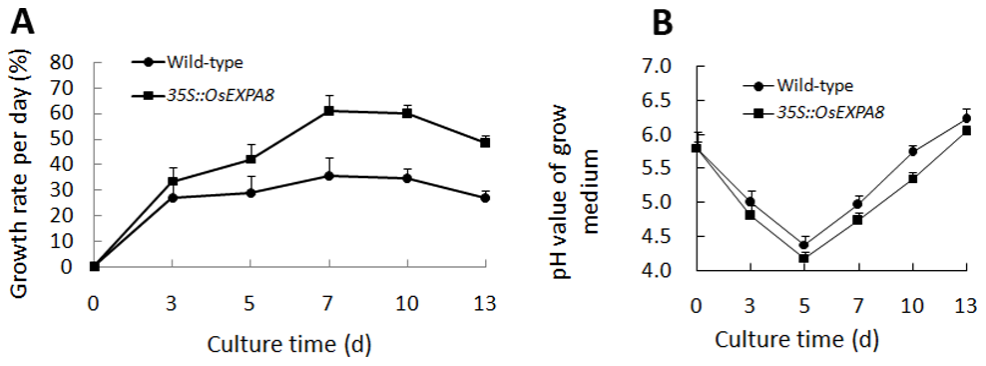
The growth rate of *35S::OsEXPA8* suspension cells. (A) pH value of growth medium, (B) The growth rate of suspension cells per day for *35S:OsEXPA*8 transgenic line 1 (L1) and wild-type cultures monitored with the passage of time in culture. WT: wild-type. Values are the means of three biological replications ± standard error.

The changes in the growth of suspension-cultured cells of *35S::OsEXPA8* transgenic line 1 (L1) were depicted in Figure 7. Three distinct phases were apparent in wild-type and *35S::OsEXPA8* cultures: a slow growth phase until day 5, then a rapid, presumably exponential phase, between days 5 and 10, and finally a stationary phase during days 10–13. Compared with wild-type cultures, the average growth rate per day of transgenic cells increased drastically (Figure 7A). The growth of transgenic cell cultures began to display significant superiority from day 5, and its rapid growth continued until day 10. The maximum growth increment per day of transgenic suspension cells was up to 61.08%, compared to approximately 35.43% of wild-type suspension cells.

Plant cells grown in suspension culture can regulate the external pH during the growth cycle by the activation of the plasma membrane H^+^-ATPase [2]. Figure 7B displayed the pH alteration of the growth medium during the cell culturing. For both transgenic and wild-type growth medium, the pH values decreased drastically from day 0 to day 5. The pH value of growth medium for transgenic cell cultures decreased steeply to the minimum on day 5 (4.18) and then increased progressively to 6.05 on day 13. Comparatively, the pH value of growth medium for wild-type cells decreased to 4.38 on day 5 and reached the maximum of 6.22 on day 13. In the exponential phase, different from increasing progressively from 4.18 (day 5) to 5.35 (day 10) for transgenic cell cultures, the pH value for wild-type cell cultures dramatically increased from 4.38 (day 7) to 5.74 (day 10). It has been reported that capacity of the cell wall to expand is believed to be influenced by pH [3], [25], and low pH activates expansins, which facilitating cell wall extension [3]. Based on these quantitative analyses, it is reasonable to conclude that overexpression of *OsEXPA8* increased the growth of suspension-cultured cells, which is also reflected by the short-term pH value of the medium.

### 4. Overexpression of *OsEXPA8* Enhances Cell Wall Extensibility of Suspension Cells

Expansins are cell wall-loosening proteins that mediate pH-dependent wall loosening [26], and are involved in many physiological processes in plants [3]. Expansion of the cell wall is an essential component of growth, and cell wall mechanical properties play a key role in plant growth. At the macroscopic level, the elastic moduli of plant cell walls of different compositions have been determined mainly by tensile or bending tests [27], [28]. Using AFM technology, it is now feasible to characterize the nanomechanical properties of living cells under near-physiological conditions [29], and recently the technique has been used to measure the living cell wall stiffness of Arabidopsis [22]. Here, we used AFM technology to explore cell wall stiffness alteration caused by *OsEXPA8* overexpression.

**Figure 8:**
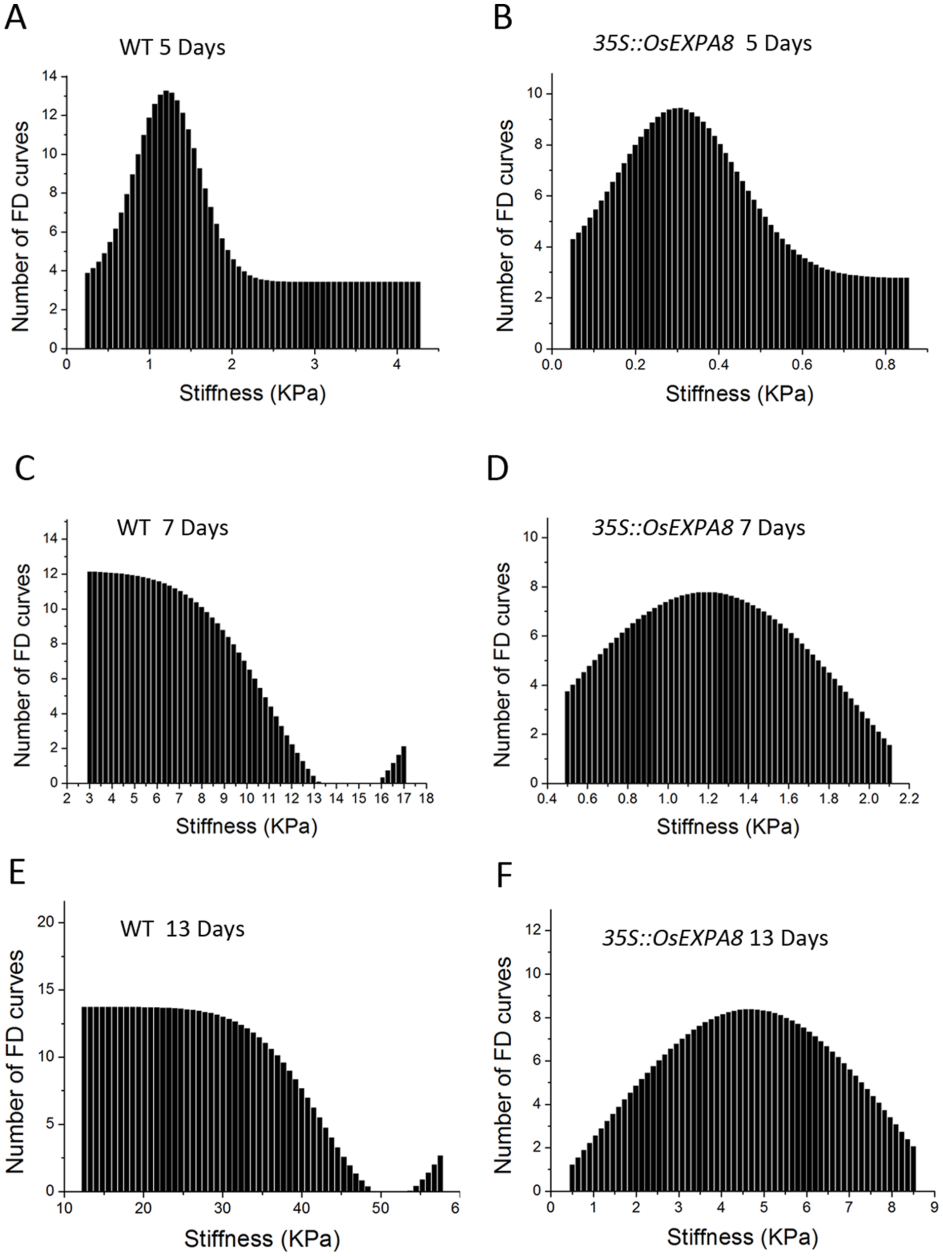
Cell wall stiffness profiles of wild-type and *35S*::*OsEXPA8* suspension cells with the passage of time in culture. (A) and (B) on day 5, (C) and (D) on day 10, (E) and (F) on day 13. Each graph corresponds to stiffness values of 150 FD curves obtained on 30 cells, which derived from three biological replications from *35S::OsEXPA*8 transgenic line 1. WT: wild-type.

Cell wall stiffness was measured as a function of the phase of growth and compared between wild-type (Figure 8A, C and E) and *35S::OsEXPA8* transgenic line 1 suspension-cultured cells (Figure 8B, D and F). In the cell suspensions, cells of different size coexist. In order not to be influenced by the cell size, cells of different sizes (small, medium and large) were used for the measurements and the desired tip penetration location in the cell wall surface is right above the nucleus. In Figure 8, stiffness distributions on the cell wall are represented graphically in the form of histograms which display the number (y axis) of pixels (i.e., FD curves) that have a given stiffness (x axis) in a force-volume data file. During the growth period from days 0 to 13, the average stiffness for both wild-type and transgenic suspension cells increased (Figure 8). After five days of culturing, the average stiffness of the cell wall was fairly low, 2.32 KPa for wild-type and 0.35 KPa for transgenic suspension cells (Figure 8A and B). From day 5 to 10, the average stiffness for both wild-type and transgenic cells are increased (7.36 KPa for wild-type and 1.22 KPa for transgenic cells) (Figure 8C and D). The average stiffness of wild-type cells showed a significant rise (27.08 KPa) (Figure 8E) with comparison of that for transgenic suspension cells (4.65 KPa) (Figure 8F). During the whole growth period, the average wall stiffness for wild-type suspension cells is over six-fold than that for the transgenic suspension cells. The variance analysis demonstrated that the average stiffness value of transgenic cells on days 5, 10 and 13 were significantly lower than the counterparts of wild-type cells, respectively (Figure S1). Since neither the location of tip penetration nor the size of the cells influenced the patterns of stiffness distribution, the phenomenon can be deemed to be governed exclusively by OsEXPA8 levels.

### 5. Overexpression of *OsEXPA8* Affects Cell Wall Compositions

How expansins cause the loosening and extension of the cell wall remains unclear, while it is thought that expansins dissociate matrix polysaccharides from cellulose [3] and change cell wall compositions. It has been proposed that, during cell rapid growth, a continuous production and deposition of wall components (xyloglucan, cellulose, and pectic polysaccharides) is required to furnish the structural conditions necessary for a rapid expansion of the cell wall [2], [3]. The walls of growing plant cells are characterized by high synthetic rates and a selective turnover of polysaccharides, both of which facilitate their expansion [30]. The presence of lignin or polysaccharides on the surface of the cells in suspension cultures has been noted [22], [31], [32]. The ratio of lignin/polysaccharide peaks in a FTIR spectrum has been considered to estimate lignin content relative to the content of polysaccharides in the cell wall [33], which determines the cell growth [22]. The average and area-normalized FTIR absorbance spectra from 1800 to 900 cm^−1^ is thought to reflect the relative changes in the main biochemical components of plant cell wall [9]. The bands at 1160 cm^−1^ (C-O-C vibration), 1425 cm^−1^ (C-H stretch), and 1540 cm^−1^ are assigned to polysaccharides [34], [35], cellulose [34], [36] and lignin [22]. The band at 1740 cm^−1^ derives from the C = O bond in ester groups [35], [36] and is characteristic of polysaccharides.

**Figure 9:**
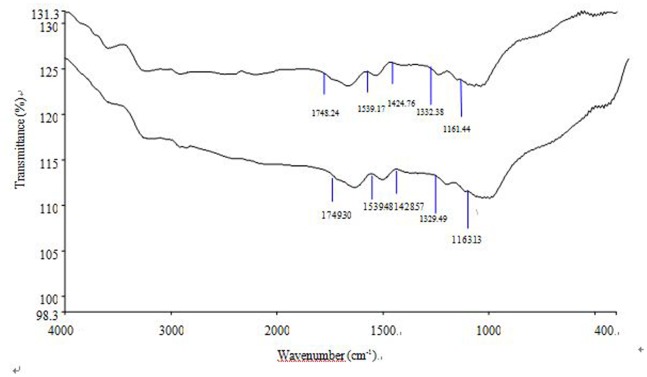
Behavior of infrared absorption bands in cell wall isolates. Cell wall isolates of wild-type and *35S::OsEXPA8* transgenic line 1 suspension cells on day 10 was used for wall composition analysis. Average and area-normalized Fourier-transform infrared (FTIR) spectra of isolated cell walls from wild-type (lower) and *35S::OsEXPA8* (upper) line 1 suspension cells.

Figure 9 illustrated the FTIR spectra of the cell wall from wild-type and *OsEXPA8* transgenic suspension cultures. Interestingly, in *35S*::*OsEXPA8* transgenic cells, the 1150–950 cm^−1^ absorption bands appeared strongly modified in shape, suggesting that the relative polymer levels in these plants had changed. According to the FTIR spectra of suspension cell walls on day 10, both lignin and polysaccharides (cellulose, xyloglucan, and pectic polysaccharides) were present in the cell walls (Figure 9). The ratios of 1540/1160 cm^−1^, 1540/1425 cm^−1^, and 1540/1740 cm^−1^ are used to estimate lignin content relative to that of polysaccharide, cellulose and esters (polysaccharide origin), respectively, in the cell wall. Compared to counterparts of wild-type suspension cells, the values of all three ratios from walls of *35S::OsEXPA8* transgenic suspension cells significantly decreased, which reflected an increase in the content of polysaccharides relative to that of lignin (Table 2).

**Table 2:** Height ratios of characteristic lignin and carbohydrate FTIR peaks in isolated cell walls from wild-type and *35S::OsEXPA8* transgenic suspension cells.

		Wild-type	L1	L4	L5
FTIR peakratios	*I*_1540/1160_	1.00±0.03	0.96±0.01*	0.94±0.02*	0.95±0.01*
	*I*_1540/1425_	0.95±0.01	0.91±0.01*	0.89±0.01*	0.92±0.03*
	*I*_1540/1740_	1.01±0.04	0.91±0.04*	0.93±0.05*	0.90±0.02*

Suspension cultures on day 10 from three independent *35S::OsEXPA8* transgenic lines (L1, L4 and L5) were used for preparing cell wall extracts, respectively. Values are the means of three biological replications ± standard error. Asterisks (*) indicate *35S::OsEXPA8* transgenic suspension cell parameters were significantly different from that of wild-type cells by statistical analysis using the Student’s *t*-test program (*P*<0.01).

Peak assignations: 1160 cm^−1^, C-O-C vibration of the glycosidic link in cellulose, xyloglucan, or pectic polysaccharides; 1425 cm^−1^, C-H stretch in CH_2_ of cellulose; 1540 cm^−1^, lignin aromatic ring stretching; 1740 cm^−1^, C = O stretch in ester groups.

## Discussion

### 1. Constitutive Expression of *OsEXPA8* Increases Plant Height and Leaf Size

*OsEXPA8* overexpression resulted in morphological changes in the root system architecture, plant height, and leaf length and width (Figure 3 and 4). Analysis of the cell size in leaf and root vascular bundles showed that *OsEXPA8* overexpression induced the cell enlargement (Figure 5 and 6), and also enhanced the growth of suspension cells (Figure 7A). Several previous studies have shown a correlation between the abundance of expansins and plant height [1], [8], [9]. Rice plants transformed with sense and antisense *OsEXP4* constructs also showed corresponding changes in terms of the coleoptile elongation [15], and the ectopic expression of *PttEXPA1* in hybrid aspen increased stem internode elongation and leaf growth through promoting cell wall expansion in primary and secondary tissues [37]. The altered morphology reported in this study, together with developmental alterations described in other plant species, supports the hypothesis that expansins may have a role in the specification of plant architecture.

### 2. Exploration of the Cell Wall Nanomechanics by AFM Technology is a Novel Model for Measuring Cell Wall Extensibility

During plant growth, the plant cell wall must be sufficiently strong to equilibrate the high turgor but pliable enough to permit cell enlargement [38], [39]. Expansins are cell wall proteins required for cell enlargement and cell wall loosening during many developmental processes. Up- and down-regulated expansin expression significantly affected expansin activity, leading to changes in the cell extension capacity and plant pleiotropic phenotypes [9], [15], [16], [40]. Expansin activity of excised tissues was usually detected by the extensometer [15], [38]. However, very little is known regarding the cell wall nanomechanics of living cells under near-physiological conditions. The expansion capacity of living cells may be surveyed accurately by AFM technology for the cell wall stiffness [22]. The cell wall stiffness has been proposed to play a major role in control of the cell expansion rate, with low values being proposed as a precondition for cell growth and cell wall expansion [2], [30], [38]. In our study, AFM technology was used to probe the difference of cell wall stiffness between wild-type and *35S*::*OsEXPA8* transgenic suspension cells. For the first time as we have known, AFM technique is used to investigate the wall mechanical characteristics of living single cell in rice. As we have expected, the reduction of wall stiffness for *35S::OsEXPA8* suspension cells was observed in the growth (Figure 8), which is correlated with changes of the cell wall composition revealed by FTIR spectroscopy (Table 2). The reduced stiffness of cell wall indicated the increased cell wall extension capacity, which showed a consistent correlation with increased plant growth (Figure 3 and 4), cell size (Figure 5 and 6) and cell growth (Figure 7).

Capacity of the cell wall to expand is believed to be influenced by pH [3], [25]. A striking example of this is seen at the onset of root hair formation in Arabidopsis, when a small patch of the outer epidermal cell wall becomes acidified by the local activation of H^+^-ATPases [41]. Another example is that the H^+^-ATPase activity peaks during the most rapid phase of fibre elongation [42]. Low pH activates expansins, which catalyze stress relaxation and extension of cell walls [3]. It has been reported that the elastic modulus of cultured tomato cell walls is at its lowest at the optimum pH for expansin activity [43], suggesting putative relations between the cell wall stiffness and expansin activity. In this study, this parameter of pH value was monitored with the passage of time in culture (Figure 7B), and the result indicated that changes of pH values were correlated with the alteration of cell wall stiffness. All these data demonstrate that changes of mechanical characteristics in the cell wall may be a regarded as an indicator for expansin activity in higher plants.

### 3. Changes of the Cell Wall Composition Indicate that Expansins may Promote Wall Remodeling During Cell Extension

Lignin is believed to modulate the elastic properties of the cell wall via its interaction with other constituents [44], [45], and the ratios between the lignin content relative to that of polysaccharide has been used to estimate elastic properties of the cell wall [22]. On the basis of the FTIR spectroscopy data (Table 2), the lignin is present on walls of suspension cells on day 10. In fact, the presence of lignin on suspension cells was also confirmed by phloroglucinol-staining (Figure S2). Compared to wild-type cells (Figure S2A), less lignin is present in *35S::OsEXPA8* transgenic suspension cells (Figure S2B). These results indicated that increased *OsEXPA8* levels changed cell wall compositions, which is consistent with the results found in transgenic *Petunia hybrid* plants overexpressing *PhEXPA1* and tobacco plants overexpressing *ClEXPA1* and *ClEXPA1* [8], [9].

Expansins might modulate cell wall compositions via two pathways. Firstly, a constant production and deposition of cell-wall components is needed to expand for cell rapid growth [30]. An increase of cellulose and xyloglucan content in the exponential phase of division were previously observed in suspension cultures of a dicots [46]. It has been proposed that the polysaccharides not only undergo structural transformations but are also newly produced during the expansion and stationary phases of growth [22]. In addition, cell walls of grasses are loaded with ferulic acid during lignifications, and expansins may be involved in regulating the process of ferulic acids cross-linked into lignins. How the expansin gene mediates lignin accumulation merits further investigation. In *OsEXPA8* overexpression transgenic suspension cells, the ratios of lignin/polysaccharide content decreased (Table 2). Therefore, the available evidence demonstrated that expansins take part in the assembly of the cell wall by changing the polymer composition of cell walls and the manner of polymer interactions in expanding tissues.

In summary, we provide direct evidence that overexpression of the root-specific α-expansin gene *OsEXPA8* increases root length and plant height by stimulating cell growth. Enhancement in the cell length of root and leaf vascular bundles and growth rate of suspension cells provides essential information of the cellular basis for expansin-mediated plant growth regulation. Modification of cell wall compositions mediated by *OsEXPA8* overexpression changed the wall mechanical properties, which elucidated the cell loosening process both biochemically and mechanically.

## Supporting Information

Figure S1 **Comparison of the average stiffness of the cell wall between wild-type and** ***35S::OsEXPA8*** **transgenic cells with the passage of time in culture.**
(TIF)

Figure S2 **The presence of lignin in the walls of suspension cells of wild-type (A) and** ***35S::OsEXPA8*** **transgenic line1 (B) on day 10.** Suspension cells were stained with 1% phloroglucinol for 1 min and then 25% hydrochloric acid for 30 min, and were observed by stereoscope microscopes. Scale bars: 200 µm.
(TIF)
