# Supplementary figures and images for: Overexpression of OsEXPA8, a Root-Specific Gene, Improves Rice Growth and Root System Architecture by Facilitating Cell Extension
Source: PLoS One. 2013 Oct 4;8(10):e75997. doi: 10.1371/journal.pone.0075997 (PMC3790854; doi:10.1371/journal.pone.0075997)

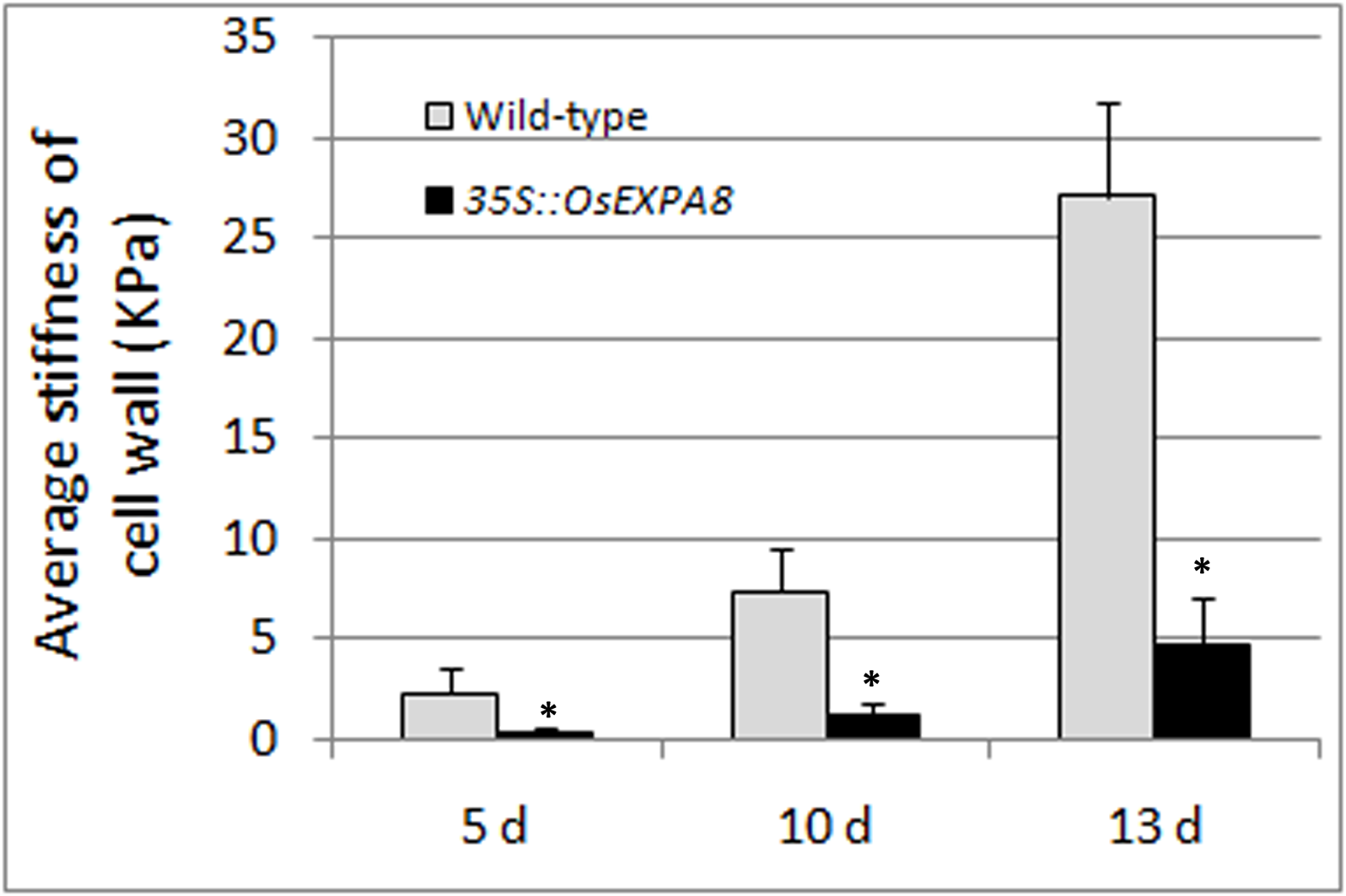

Supplement: Figure S1 — Comparison of the average stiffness of the cell wall between wild-type and 35S::OsEXPA8 transgenic cells with the passage of time in culture. (TIF) [file pone.0075997.s001.tif]

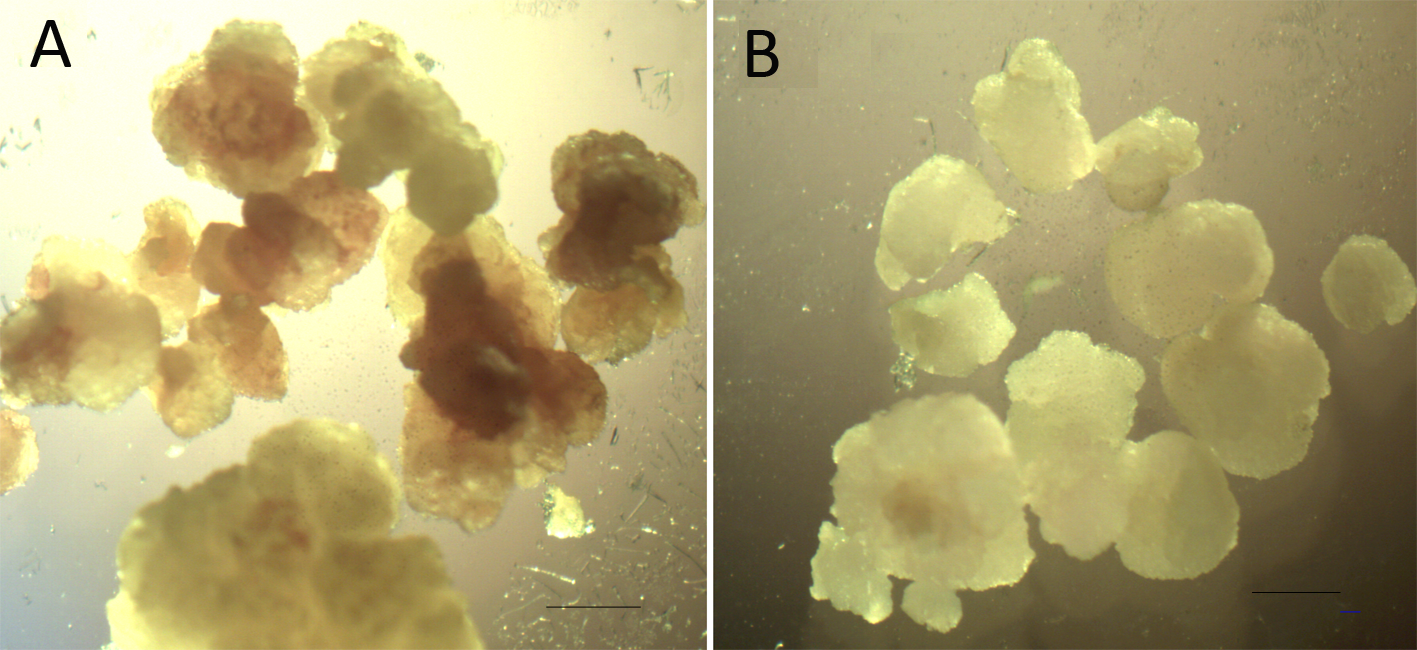

Supplement: Figure S2 — The presence of lignin in the walls of suspension cells of wild-type (A) and 35S::OsEXPA8 transgenic line1 (B) on day 10. Suspension cells were stained with 1% phloroglucinol for 1 min and then 25% hydrochloric acid for 30 min, and were observed by stereoscope microscopes. Scale bars: 200 µm. (TIF) [file pone.0075997.s002.tif]
